# Perioperative Hemodynamic Responses in Hypertensive Patients Undergoing Oral Surgery and Development of the Perozo Protocol

**DOI:** 10.7759/cureus.107163

**Published:** 2026-04-16

**Authors:** Jenny Perozo-Quiroz, David J Perozo Gonzalez, David Beltran, Syed A. A Rizvi, Marcos Sanchez-Gonzalez

**Affiliations:** 1 Oral Surgery, La Universidad del Zulia, Maracaibo, VEN; 2 Interventional Cardiology, Venezuelan Association of Cardiology, Caracas, VEN; 3 Periodontics, Beltran Periodontics, Kissimmee, USA; 4 Biomedical Sciences, Larkin University, Miami, USA; 5 Health Services, Lake Erie College of Osteopathic Medicine, Bradenton, USA

**Keywords:** anxiety, arterial hypertension, circadian rhythm, hemodynamic monitoring, local anesthetics, oral surgery, perioperative cardiovascular risk, safety patient

## Abstract

Background

Arterial hypertension (AH) is a highly prevalent systemic condition affecting approximately one-third of adults worldwide and is commonly encountered in patients undergoing oral surgical procedures. Despite its clinical significance, perioperative cardiovascular management in hypertensive dental patients remains inconsistently standardized. The Perozo protocol is a structured three-phase perioperative management framework proposed to address this gap.

Objectives

The objective of this article was to characterize perioperative hemodynamic responses in normotensive and controlled hypertensive patients undergoing oral surgery, evaluate the influence of anesthetic selection, surgical timing, and preoperative anxiety, and propose a structured perioperative management framework (the Perozo protocol).

Methods

This study represents a retrospective analysis of prospectively collected, de-identified observational clinical data from patients undergoing oral surgical procedures at two public hospitals in Maracaibo, Venezuela. Systolic blood pressure (SBP), diastolic blood pressure (DBP), mean arterial pressure (MAP), and heart rate (HR) were recorded at four predefined perioperative time points: (1) preoperative baseline, obtained after rest prior to the procedure; (2) during local anesthetic administration, immediately following injection; (3) active intraoperative phase, during surgical manipulation; and (4) immediate postoperative period, shortly after completion while the patient remained in the dental chair. Preoperative anxiety was assessed using the State-Trait Anxiety Inventory (STAI). Local anesthetic regimens included 2% lidocaine with 1:100,000 epinephrine or 3% plain mepivacaine. Procedures were stratified by timing (morning vs. afternoon). Statistical analyses included repeated-measures ANOVA, Pearson Chi-squared testing, and Pearson correlation coefficients (α=0.05).

Results

Thirty-nine patients were included: normotensive (n=21) and hypertensive receiving pharmacological treatment (n=18). Baseline blood pressure status was the primary determinant of perioperative hemodynamic variability (η²=0.33). Hypertensive patients demonstrated greater systolic blood pressure elevations, with a mean preoperative SBP of 159 mmHg compared with 128 mmHg in normotensive patients. Intraoperative SBP peaks exceeded 170 mmHg in hypertensive individuals. Hemorrhagic complications occurred significantly more frequently in hypertensive patients (66.7% vs. 9.5%; Cramer's V=0.55; p=0.008). Epinephrine-containing anesthesia produced greater increases in blood pressure and heart rate compared with plain mepivacaine. Preoperative anxiety was associated with greater systolic blood pressure increases in hypertensive patients and greater heart rate responses in normotensive patients. Observational findings from this pilot cohort informed the proposed Perozo protocol; however, formal validation has not yet been performed.

Conclusions

Baseline hypertensive status is a major determinant of perioperative hemodynamic variability during oral surgery. Anesthetic selection, surgical timing, anxiety management, and structured monitoring represent modifiable risk factors. The Perozo protocol represents a pilot, hypothesis-generating perioperative management framework that may improve cardiovascular safety in hypertensive dental patients. Further multicenter prospective validation is required.

## Introduction

Arterial hypertension is the most prevalent systemic disease encountered in oral surgery practice, affecting an estimated 20%-30% of adults globally [1]. Under the stricter 130/80 mmHg guideline, approximately 48% of US adults have hypertension, compared to 33% globally using the 140/90 mmHg standard. However, 80% of hypertensive individuals worldwide do not receive adequate treatment [2]. Many of these individuals undergo dental and oral surgical procedures regularly; however, perioperative cardiovascular management in hypertensive dental patients remains poorly standardized, with considerable variability across training programs and clinical practice settings.

The oral surgical encounter introduces a combination of physiological stressors capable of destabilizing hemodynamic equilibrium in susceptible individuals. Procedural anxiety, nociceptive stimulation, vasoconstrictor-containing local anesthetics, and activation of the systemic stress response collectively stimulate the sympathoadrenal axis, resulting in acute elevations in blood pressure (BP) and heart rate (HR) [3,4]. In normotensive patients, these responses are typically transient and well-tolerated. In hypertensive patients, particularly those with suboptimal pharmacologic control, the same stimuli may precipitate intraoperative hypertensive crises, hemorrhagic complications, and acute cardiovascular events [5, 6].

Despite the clinical relevance of this risk, no universally adopted perioperative protocol currently exists to guide dental surgeons in the standardized management of hypertensive patients. Existing recommendations from the American Heart Association (AHA) and American Dental Association (ADA), as well as the World Health Organization (WHO) and International Society of Hypertension (ISH), provide general guidance regarding BP thresholds and anesthetic considerations. However, these recommendations do not offer an integrated, operationally specific perioperative framework readily adaptable to routine dental surgical practice [7-9].

The present study was designed to address this gap through an observational clinical investigation examining perioperative hemodynamic responses during oral surgery, with systematic evaluation of the modifying effects of anesthetic agent selection, surgical timing, and preoperative anxiety. The clinical findings generated by this study informed the development of the Perozo protocol, a structured 20-step, three-phase perioperative management framework aligned with international cardiovascular and dental practice recommendations.

Given the global burden of hypertension and its high prevalence in both low- and middle-income and high-income settings, the dental setting represents an underused point of contact for cardiovascular risk identification and management. A structured perioperative protocol may improve patient safety, reduce preventable adverse events, and support broader efforts to integrate oral and systemic health across clinical disciplines [10].

The Perozo protocol is proposed as a structured, clinically adaptable framework derived from observational findings to address this gap.

## Materials and methods

Study design and setting

This study represents a retrospective analysis of prospectively collected clinical data obtained from patients undergoing oral surgical procedures at two public hospitals in Maracaibo, Venezuela. The original data collection was conducted with institutional authorization from the Bioethics Committees of the Hospital Universitario de Maracaibo and the Hospital Central de Maracaibo in accordance with the Declaration of Helsinki.

For the purposes of the present analysis, all data were fully de-identified prior to investigator access, and no direct or indirect identifiers were available to the research team. A formal determination issued by the Lake Erie College of Osteopathic Medicine Institutional Review Board classified this study as not involving human subjects research based on the use of de-identified data; therefore, additional IRB review and informed consent were not required.

No interventions were assigned for research purposes, and all treatments reflected routine clinical decision-making. Accordingly, this study does not meet the definition of a clinical trial under the International Committee of Medical Journal Editors (ICMJE) criteria.

Although the source data were originally recorded prospectively during routine clinical care, the present study is a retrospective secondary analysis of fully de-identified data, and no interventions were assigned for research purposes.

Study population

A total of 39 patient records meeting eligibility criteria were included in the analysis and categorized into two groups: normotensive (n=21) and hypertensive patients receiving stable pharmacological treatment (n=18). Hypertensive patients were defined as those with an established diagnosis of primary arterial hypertension and documented blood pressure control at the time of data collection. Inclusion criteria consisted of adult patients who underwent oral surgical procedures and had complete perioperative hemodynamic data available. Exclusion criteria included uncontrolled hypertension (SBP≥180 mmHg or diastolic blood pressure (DBP)≥110 mmHg at baseline), history of acute cardiovascular events within the preceding three months, pregnancy, and contraindications to the anesthetic agents used.

Hemodynamic monitoring

Systolic blood pressure (SBP), diastolic blood pressure (DBP), mean arterial pressure (MAP), and heart rate (HR) were recorded at four standardized perioperative time points: (1) preoperative baseline, (2) during local anesthetic administration, (3) during the active intraoperative phase, and (4) in the immediate postoperative period. Measurements were obtained using automated digital sphygmomanometers of the same make and specification available within the participating institutions. All devices were routinely maintained and calibrated according to institutional biomedical engineering protocols to ensure measurement consistency. The present study represents a secondary analysis of fully de-identified clinical data; therefore, individual informed consent for this analysis was not required, as determined by institutional review.

Anxiety assessment

Preoperative anxiety was assessed using the State-Trait Anxiety Inventory (STAI), adapted for the clinical dental population by Spielberger and Díaz Guerrero, and classified as low, moderate, or high according to validated scoring thresholds [11,12]. The STAI was administered immediately before the surgical procedure. Use of the STAI was conducted under appropriate licensing obtained from Mind Garden, Inc., and the instrument was administered in accordance with the official STAI manual and publisher guidelines. The instrument was used solely as a standardized measurement tool following the methodology described in the original STAI manual and validation studies. No questionnaire items, scoring sheets, or copyrighted STAI materials are reproduced in this manuscript.

For interpretative purposes, STAI scores were categorized into three clinically relevant levels based on commonly reported thresholds in validation studies. Lower scores correspond to minimal anxiety, mid-range scores indicate moderate anxiety, and higher scores reflect elevated anxiety levels. In general clinical application, scores in the lower range (approximately ≤37) are considered indicative of low anxiety, scores in the mid-range (approximately 38-44) reflect moderate anxiety, and scores in the higher range (≥45) are associated with high anxiety. These thresholds were used to facilitate standardized classification of patient anxiety levels while adhering to the original STAI scoring methodology without reproducing proprietary materials.

Anesthetic agents

Local anesthetic agents administered during routine clinical care included 2% lidocaine with 1:100,000 epinephrine and 3% plain mepivacaine without vasoconstrictor. The selection of anesthetic agent was determined by the operating surgeon based on individual patient cardiovascular risk assessment and standard clinical practice, consistent with American Heart Association (AHA)-American Dental Association (ADA) recommendations, including limitation of epinephrine dosage in patients with hypertension. No randomization, protocol-driven allocation, or investigator-directed intervention was performed. Anesthetic use was observed as part of routine care and subsequently analyzed in relation to perioperative hemodynamic outcomes.

Surgical timing

Procedures were stratified by scheduling as morning (08:00-12:00) or afternoon (13:00-17:00) shifts to account for circadian variations in blood pressure, including the morning surge and diurnal fluctuations, which may influence perioperative hemodynamic responses.

Statistical analysis

Repeated-measures ANOVA was used to evaluate hemodynamic changes across the four perioperative phases within and between groups. Pearson chi-square testing was used to assess associations between categorical variables, and the strength of association was quantified using Cramer's V. Pearson correlation coefficients were used to examine relationships between anxiety scores and hemodynamic parameters. A two-tailed significance threshold of α=0.05 was applied throughout

## Results

A total of 39 patients undergoing oral surgical procedures were included in the analysis, comprising 21 normotensive patients and 18 hypertensive patients receiving pharmacologic treatment. Baseline blood pressure status was identified as the primary determinant of perioperative hemodynamic variability across procedural phases. Repeated-measures analysis of variance demonstrated a statistically significant effect of hypertension status on perioperative systolic blood pressure responses (F=8.41, p=0.006). These findings indicate that hypertensive patients experienced greater hemodynamic variability and higher peak blood pressure values during oral surgical procedures compared with normotensive individuals.

Based on observational findings from this pilot cohort and in alignment with current AHA-ADA and WHO-ISH recommendations, a structured perioperative management framework, designated the Perozo Protocol, was proposed as a hypothesis-generating model. This framework organizes perioperative cardiovascular risk management into three sequential phases: preoperative, intraoperative, and postoperative, as illustrated in Table 1.

**Table 1:** Integrated Perozo protocol outlining perioperative cardiovascular risk stratification, emergency pharmacologic management, and postoperative care in hypertensive patients undergoing oral surgery SBP - systolic blood pressure; DBP - diastolic blood pressure; SL - sublingual; NSAIDs - nonsteroidal anti-inflammatory drugs AHA-ADA; WHO-ISH guidelines are referenced in this table. Additional consideration: epinephrine should be limited to 0.04 mg (two cartridges of 1:100,000) in controlled hypertensive patients. This protocol was proposed by Perozo Quiroz JK, La Universidad del Zulia, Venezuela in a retrospective analysis of prospectively collected observational clinical data (n=39).

Phase	Category	Parameter / Iintervention	Details / threshold	Clinical action / indication
Phase I	Risk stratification	Low risk	SBP < 140 mmHg; DBP < 90 mmHg	Proceed with routine procedure using standard precautions
		Moderate risk	SBP 140–159 mmHg; DBP 90–99 mmHg	Minor procedures; reduce anxiety; monitor BP
		High risk	SBP 160–179 mmHg; DBP 100–109 mmHg	Short procedures with strict BP monitoring
		Very high risk	SBP ≥ 180 mmHg; DBP ≥ 110 mmHg	Defer procedure; obtain urgent medical consultation
Phase II	Emergency medications	Captopril 25 mg (SL)	—	Hypertensive crisis
		Nifedipine (bitten capsule)	—	Acute blood pressure surge
		Nitroglycerin (SL)	—	Coronary symptoms
		Clonidine	—	Refractory hypertension
Phase III	Postoperative care	Vital signs monitoring	During recovery	Monitor hemodynamic stability
		Analgesic selection	Avoid NSAIDs	Reduce cardiovascular risk
		Antibiotic therapy	If indicated	Based on clinical judgment
		Discharge instructions	Written guidance	Ensure patient understanding
		Warning signs	Hemodynamic symptoms	Educate patient on red flags
		Follow-up	Structured protocol	Ensure continuity of care

**Figure 1:**
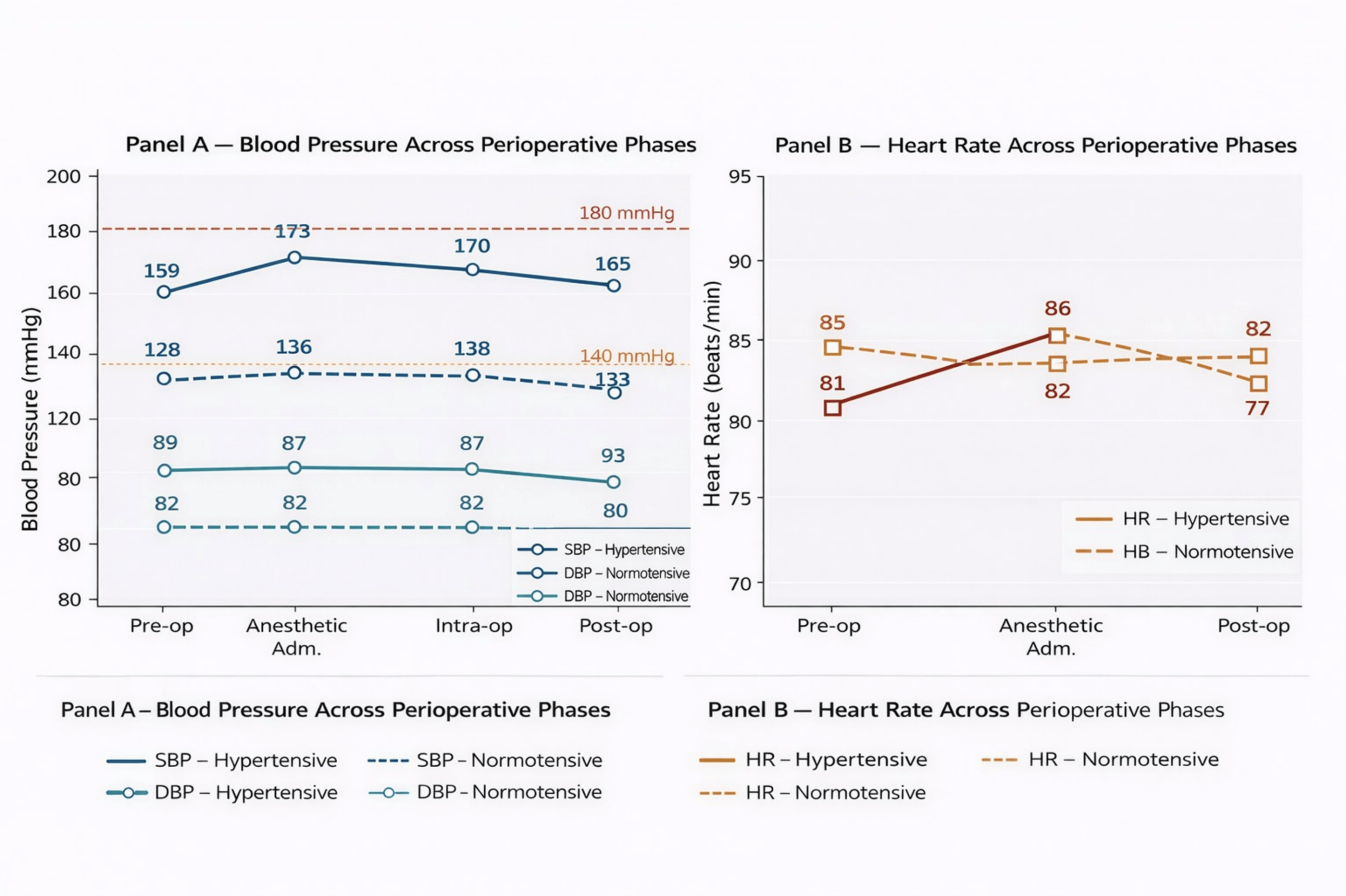
Perioperative hemodynamic responses in hypertensive and normotensive patients undergoing oral surgery SBP - systolic blood pressure; DBP - diastolic blood pressure; HR - heart rate (A) Systolic and diastolic blood pressure changes across perioperative phases. (B) Heart rate responses during the same perioperative phases.

Hypertensive patients demonstrated substantially higher systolic blood pressure values throughout the perioperative period. Mean preoperative systolic blood pressure was 159 mmHg among hypertensive patients compared with 128 mmHg in normotensive patients. During the intraoperative phase, systolic blood pressure reached peak values of approximately 170 mmHg in hypertensive patients, whereas normotensive patients maintained systolic blood pressure values between 136 mmHg and 138 mmHg across the perioperative phases. Diastolic blood pressure followed a similar pattern, reaching 97 mmHg intraoperatively in hypertensive patients compared with 82 mmHg in normotensive patients. Heart rate responses were comparable between groups, with hypertensive patients reaching a peak heart rate of 85 beats per minute and normotensive patients reaching 86 beats per minute during the intraoperative phase. These perioperative hemodynamic differences between hypertensive and normotensive patients are summarized in Table 2 and illustrated graphically in Figure 1, where Figure 1A demonstrates systolic and diastolic blood pressure changes across perioperative phases and Figure 1B depicts heart rate responses.

**Table 2:** Comparison of perioperative hemodynamic and clinical outcomes between hypertensive and normotensive patients undergoing oral surgery SBP - systolic blood pressure; DBP - diastolic blood pressure; HR - heart rate

Variable	Normotensive (n = 21)	Hypertensive (n = 18)	Statistical test	Test statistic	p-value
Preoperative SBP (mmHg)	128 ± 9	159 ± 12	Repeated-measures ANOVA	F=8.41	0.006
Intraoperative SBP (mmHg)	136 ± 10	170 ± 15	Repeated-measures ANOVA	F=9.02	0.004
Intraoperative DBP (mmHg)	82 ± 7	97 ± 9	Repeated-measures ANOVA	F=6.75	0.013
Peak HR (beats/min)	86 ± 8	85 ± 9	Repeated-measures ANOVA	F=0.14	0.71
Hemorrhagic complications (%)	9.50%	66.70%	Pearson Chi-squared	χ²=7.02	0.008
Anxiety score vs SBP change	—	—	Pearson correlation	r=0.58	0.01

**Figure 2:**
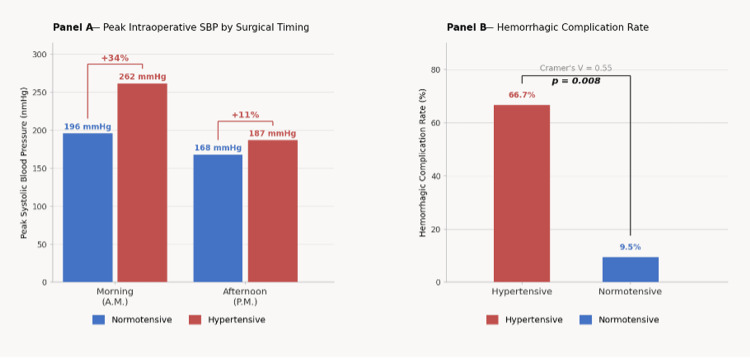
Effect of hypertension status and surgical timing on perioperative outcomes (A) Peak intraoperative systolic blood pressure during morning and afternoon procedures. (B) Hemorrhagic complication rates in hypertensive versus normotensive patients.

Hemorrhagic complications occurred significantly more frequently in hypertensive patients than in normotensive individuals. Specifically, hemorrhagic complications were observed in 66.7% of hypertensive patients compared with 9.5% of normotensive patients. Pearson chi-square analysis demonstrated a significant association (χ²=7.02, p=0.008), with a large effect size (Cramer's V=0.55). These findings are also summarized in Table 2. These results suggest that elevated baseline blood pressure is an important determinant of procedural bleeding risk during oral surgery. The relationship between hypertension status, peak intraoperative systolic blood pressure, and hemorrhagic complications is illustrated in Figure 2, where Figure 2A shows peak intraoperative systolic blood pressure values according to surgical timing and Figure 2B presents hemorrhagic complication rates in hypertensive versus normotensive patients.

The type of local anesthetic administered also influenced perioperative hemodynamic responses. Lidocaine 2% with epinephrine (1:100,000) produced greater elevations in blood pressure and heart rate compared with plain 3% mepivacaine, particularly among hypertensive patients. The sympathomimetic effects of epinephrine, combined with procedural anxiety and nociceptive stimulation, contributed to transient hypertensive peaks and mild tachycardia. In normotensive patients, vasoconstrictor-containing anesthesia was generally well tolerated and did not produce clinically significant hemodynamic instability.

Surgical timing demonstrated a measurable influence on perioperative blood pressure responses. Morning procedures (08:00-12:00) were associated with higher peak systolic blood pressure values than afternoon procedures (13:00-17:00), consistent with established circadian blood pressure variation. Hypertensive patients undergoing morning procedures reached peak systolic blood pressure exceeding 250 mmHg compared with 196 mmHg in normotensive patients. In afternoon procedures, hypertensive patients reached peak systolic blood pressure values of 187 mmHg compared with 168 mmHg in normotensive patients. The magnitude of reduction in peak systolic blood pressure from morning to afternoon procedures was greater among hypertensive patients, suggesting enhanced circadian variability in this group, as illustrated in Figure 2A.

**Figure 3:**
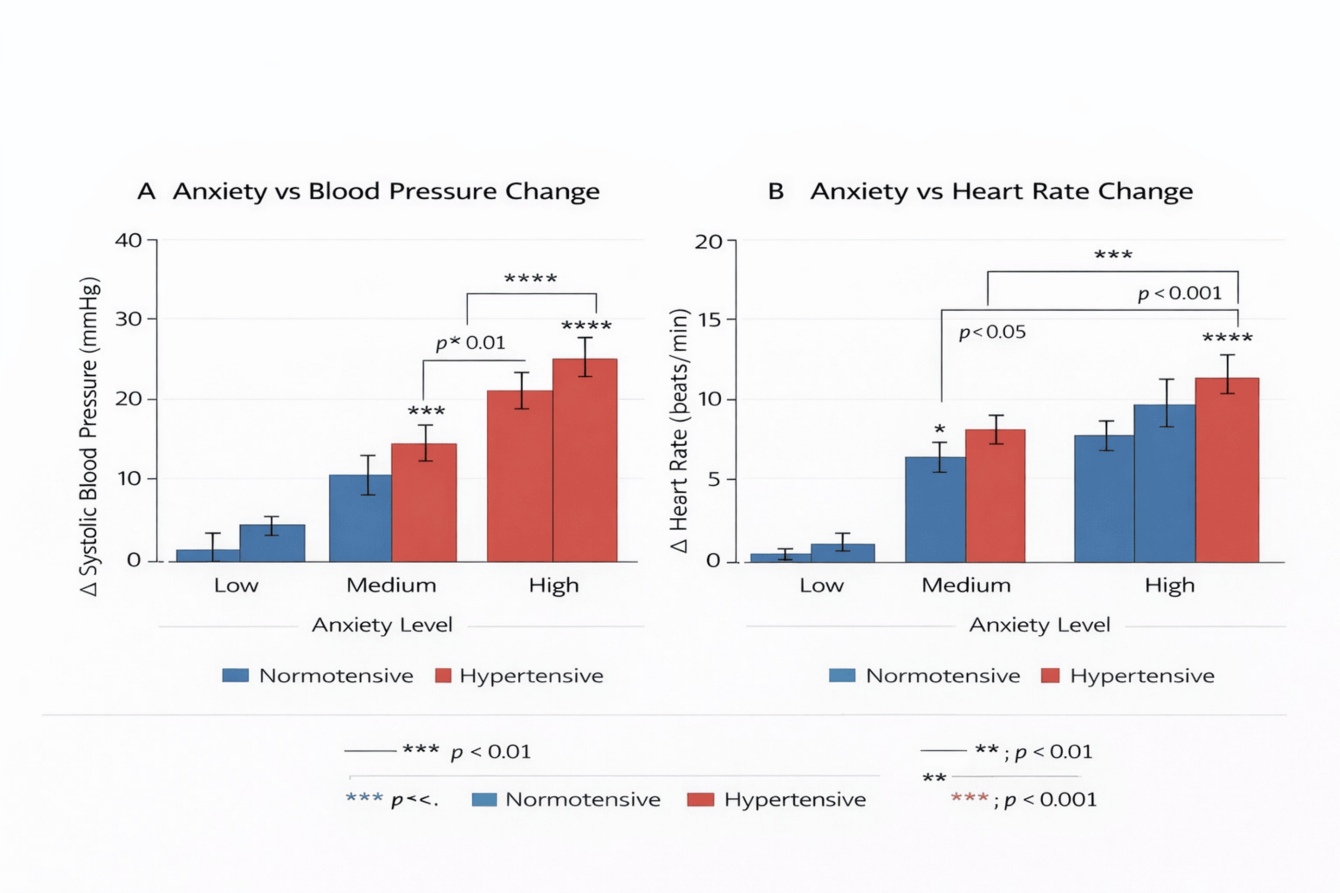
Differential anxiety-mediated hemodynamic responses by cardiovascular status in patients undergoing oral surgery (A) Systolic blood pressure changes across anxiety levels. (B) Heart rate responses according to anxiety classification.

Preoperative anxiety levels were also associated with differential hemodynamic responses depending on baseline cardiovascular status. In hypertensive patients, increasing anxiety levels were primarily associated with elevations in systolic blood pressure. Mean systolic blood pressure increases rose from 1.5 ± 2.0 mmHg at low anxiety levels to 14.7 ± 2.5 mmHg at moderate anxiety levels and 24.9 ± 2.8 mmHg at high anxiety levels. In contrast, normotensive patients demonstrated smaller systolic blood pressure changes across anxiety categories but exhibited a more pronounced chronotropic response. Heart rate increased from 1.0 ± 0.6 beats per minute at low anxiety levels to 6.5 ± 1.0 beats per minute at moderate anxiety levels and 9.6 ± 1.5 beats per minute at high anxiety levels. These anxiety-related hemodynamic responses are illustrated in Figure 3, where Figure 3A demonstrates systolic blood pressure responses across anxiety levels and Figure 3B shows heart rate responses.

## Discussion

The primary objective of this study was to characterize perioperative hemodynamic responses in hypertensive versus normotensive patients undergoing oral surgery and to identify modifiable clinical variables influencing cardiovascular stability across the procedural continuum, with the broader aim of informing a structured, hypothesis-generating perioperative management framework for dental surgical settings. Our findings indicate that baseline hypertensive status is the dominant perioperative hemodynamic determinant (η²=0.33), that hemorrhagic complication risk is substantially elevated in hypertensive patients (66.7% vs 9.5%; Cramer's V=0.55; p=0.008), and that anesthetic agent selection, surgical timing, and preoperative anxiety management each exert clinically relevant, modifiable effects on cardiovascular outcomes. Together, these observations support the rationale for a structured perioperative framework for oral surgery patients with hypertension. The Perozo protocol offers a structured and clinically applicable approach that may help reduce preventable cardiovascular complications and provides a rationale for future multicenter prospective validation in US dental surgical settings.

The Perozo protocol was proposed as a structured three-phase perioperative workflow that integrates preoperative risk stratification, intraoperative hemodynamic risk mitigation, and postoperative monitoring into a single operational framework for oral surgery. The protocol is needed because current AHA-ADA and WHO-ISH recommendations provide general guidance on blood pressure thresholds, vasoconstrictor use, and treatment deferral, but they do not offer a unified chairside protocol tailored to hypertensive dental patients. Prior literature supports several elements included in the protocol, including standardized blood pressure screening, limitation of epinephrine exposure, postponement of treatment in markedly hypertensive patients, and closer perioperative monitoring in high-risk individuals [3,5,7,9,13]. Accordingly, the Perozo protocol should be interpreted as a pilot, hypothesis-generating framework derived from observational findings and existing guideline principles, pending formal multicenter validation.

The predominance of baseline hypertensive status as the primary hemodynamic determinant is consistent with prior foundational work in the field. Muzyka and Glick [5] showed that hypertensive patients presenting to dental settings carry substantially elevated perioperative cardiovascular risk compared with normotensive peers, and our quantitative findings provide effect-size data supporting that clinical observation. Likewise, our finding that epinephrine-containing local anesthesia produces greater BP and HR elevations in hypertensive patients aligns with the systematic review by Bader et al. [3], who documented acute hemodynamic responses to vasoconstrictor-containing anesthetics across cardiovascular risk groups, reinforcing the continuing relevance of the AHA-ADA two-cartridge limit recommendation [7,13].

Our findings regarding the differential anxiety response between groups, predominantly vasopressor in hypertensive patients and more chronotropic in normotensive patients, add nuance to the dental anxiety literature, which has often treated anxiety as a unidimensional hemodynamic stressor. This pattern suggests that the physiologic substrate of anxiety-mediated hemodynamic activation may differ meaningfully between cardiovascular risk groups [14,15]. This observation contrasts with earlier work by Silvestre et al. [4], who reported comparable anxiety-related BP elevations across groups. This discrepancy may be attributable to differences in anxiety measurement instruments, patient sample characteristics, and procedural context.

The relationship between surgical timing and complication risk also warrants attention. Circadian BP variation is well established in the cardiovascular literature, with peak sympathoadrenal activity occurring in the early morning hours [16]. However, its procedural relevance in oral surgery had not been systematically quantified prior to this study [16-18]. Our findings show that morning procedures were associated with the highest peak SBP values in both groups, with hypertensive patients reaching peak systolic blood pressure values exceeding 250 mmHg compared with 196 mmHg in normotensive patients, representing a 34% difference. Afternoon procedures showed an attenuated but persistent disparity (187 mmHg vs 168 mmHg, +11%). The greater decline in peak SBP from morning to afternoon among hypertensive patients extends the existing literature by providing procedure-level outcome data stratified by circadian timing in a clinically defined high-risk population.

This study has several limitations. First, the sample size of 39 patients, while appropriate for the exploratory and descriptive aims of this investigation, limits statistical power for granular subgroup analyses and precludes definitive causal inference. Future studies with larger, adequately powered prospective samples would improve the precision of effect estimates and allow for more robust multivariable modeling of independent risk determinants.

Second, the single-country, two-center design within the Venezuelan public hospital system may limit generalizability to the United States and other high-income settings, where patterns of pharmacologic management, comorbidity burden, and clinical infrastructure may differ substantially. The prevalence of treatment-resistant hypertension, polypharmacy, and metabolic comorbidities in US hypertensive populations may further amplify perioperative risk beyond what was observed in this sample.

Third, the retrospective analysis of prospectively collected observational data introduces the possibility of unmeasured confounding from variables not captured in the original protocol, including antihypertensive medication class, duration of hypertension, target organ damage, and baseline trait anxiety, all of which should be considered in future validation studies.

From a clinical and health systems perspective, several practical implications emerge from these findings. First, mandatory preoperative BP assessment and four-level cardiovascular risk stratification, as operationalized in phase I of the Perozo protocol, could be incorporated into pre-procedural checklists in oral surgical settings, including private practices, community health centers, and academic dental programs. The findings presented here suggest that proceeding with oral surgery without documented BP control and individualized risk stratification may expose hypertensive patients to avoidable risk [19-21].

Second, dental surgical training programs at the predoctoral, postdoctoral residency, and continuing education levels may benefit from integrating structured perioperative cardiovascular risk management content into their competency frameworks. The current absence of a standardized curriculum in this area represents an important systems-level gap.

Third, the dental setting may serve as an important hypertension detection and referral touchpoint within the broader primary care ecosystem. Given that many underserved individuals have more frequent contact with dental providers than with primary care clinicians, structured BP screening and referral pathways within dental practices may offer a practical public health opportunity aligned with broader chronic disease prevention goals.

From a research standpoint, the next step is multicenter prospective evaluation of the Perozo protocol across diverse US dental surgical settings, including academic oral surgery residency programs, federally qualified health centers, and private practices serving medically complex populations. Such work would help determine generalizability, refine risk thresholds for US populations, and provide stronger evidence base for future guideline-level consideration.

## Conclusions

Arterial hypertension is a clinically significant perioperative risk factor in oral surgery that remains incompletely addressed by current practice standards. This study demonstrates that baseline hypertensive status is a major determinant of perioperative hemodynamic variability (η²=0.33), that hemorrhagic complication risk is substantially higher in hypertensive patients (p=0.008), and that anesthetic agent selection, surgical timing, and anxiety management are modifiable variables with direct relevance to cardiovascular safety outcomes.

The Perozo protocol, a structured three-phase perioperative management framework derived from observational findings in this pilot cohort and aligned with AHA-ADA and WHO-ISH recommendations, represents a proposed, hypothesis-generating approach to perioperative cardiovascular risk management in hypertensive patients undergoing oral surgery. Given the limited sample size and observational design, this framework should not be interpreted as validated and requires formal evaluation in larger, prospective multicenter studies with appropriate statistical power. Future research should prioritize multicenter prospective evaluation across diverse dental surgical settings, particularly in underserved regions where uncontrolled hypertension and limited access to coordinated oral-systemic care may compound preventable risk

## References

[1] (2026). Hypertension. https://www.who.int/news-room/fact-sheets/detail/hypertension.

[2] Virani SS, Alonso A, Aparicio HJ (2021). Heart disease and stroke statistics-2021 Update: a report from the American Heart Association. Circulation.

[3] Bader JD, Bonito AJ, Shugars DA (2002). A systematic review of cardiovascular effects of epinephrine on hypertensive dental patients. Oral Surg Oral Med Oral Pathol Oral Radiol Endod.

[4] Silvestre FJ, Verdú MJ, Sanchís JM, Grau D, Peñarrocha M (2001). Effects of vasoconstrictors in dentistry upon systolic and diastolic arterial pressure. Med Oral.

[5] Muzyka BC, Glick M (1997). The hypertensive dental patient. J Am Dent Assoc.

[6] Kaur B, Ziccardi VB (2020). Management of the hypertensive patient in dental practice. Compend Contin Educ Dent.

[7] Gupta K, Kumar S, Anand Kukkamalla M (2022). Dental management considerations for patients with cardiovascular disease-a narrative review. Rev Cardiovasc Med.

[8] Chalmers J, MacMahon S, Mancia G (1999). 1999 World Health Organization-International Society of Hypertension Guidelines for the management of hypertension. Guidelines sub-committee of the World Health Organization. Clin Exp Hypertens.

[9] Whelton PK, Carey RM, Aronow WS (2018). Guideline for the Prevention, Detection, Evaluation, and management of high blood pressure in adults: a report of the American College of Cardiology/American Heart Association Task Force on clinical practice guidelines. Hypertension.

[10] (2026). Oral health in America. https://www.nidcr.nih.gov/sites/default/files/2021-12/Oral-Health-in-America-Advances-and-Challenges.pdf.

[11] Vieco-García A, López-Picado A, Fuentes M (2021). Comparison of different scales for the evaluation of anxiety and compliance with anesthetic induction in children undergoing scheduled major outpatient surgery. Perioper Med (Lond).

[12] Caumo W, Schmidt AP, Schneider CN (2001). Risk factors for postoperative anxiety in adults. Anaesthesia.

[13] Seminario-Amez M, González-Navarro B, Ayuso-Montero R, Jané-Salas E, López-López J (2021). Use of local anesthetics with a vasoconstrictor agent during dental treatment in hypertensive and coronary disease patients. A systematic review. J Evid Based Dent Pract.

[14] Bilo G, Grillo A, Guida V, Parati G (2018). Morning blood pressure surge: pathophysiology, clinical relevance and therapeutic aspects. Integr Blood Press Control.

[15] Chellappa SL, Vujovic N, Williams JS, Scheer FA (2019). Impact of circadian disruption on cardiovascular function and disease. Trends Endocrinol Metab.

[16] Nuszkiewicz J, Rzepka W, Markiel J, Porzych M, Woźniak A, Szewczyk-Golec K (2025). Circadian rhythm disruptions and cardiovascular disease risk: the special role of melatonin. Curr Issues Mol Biol.

[17] Yao YT, Huang S, Chao M, More A (2026). The effects of daytime variation on short-term outcomes of cardiac surgical patients: a PRISMA-compliant systemic review and meta-analysis. J Cardiothorac Vasc Anesth.

[18] Gumz ML, Shimbo D, Abdalla M (2023). Toward precision medicine: Circadian rhythm of blood pressure and chronotherapy for hypertension - 2021 NHLBI workshop report. Hypertension.

[19] Yarows SA, Vornovitsky O, Eber RM, Bisognano JD, Basile J (2020). Canceling dental procedures due to elevated blood pressure: is it appropriate?. J Am Dent Assoc.

[20] Gazal G, Omar E, Alofi HA, Nassani MZ (2026). Selection of the safest local anesthetic for dental treatment in medically compromised patients: a comprehensive review. Saudi J Anaesth.

[21] Nath S, Jiang T, Barrow J, Simon L (2024). Treatment deferral for elevated blood pressure at a dental school clinic. J Dent Educ.

